# High abundances of class 1 integrase and sulfonamide resistance genes, and characterisation of class 1 integron gene cassettes in four urban wetlands in Nigeria

**DOI:** 10.1371/journal.pone.0208269

**Published:** 2018-11-29

**Authors:** Olawale Olufemi Adelowo, Therese Helbig, Camila Knecht, Franziska Reincke, Ines Mäusezahl, Jochen A. Müller

**Affiliations:** 1 Department of Environmental Biotechnology, Helmholtz Centre for Environmental Research GmbH - UFZ, Leipzig, Germany; 2 Environmental Microbiology and Biotechnology Laboratory, Department of Microbiology, University of Ibadan, Ibadan, Oyo State, Nigeria; 3 Institute of Biology/Microbiology Martin-Luther-University Halle-Wittenberg, Halle, Germany; 4 Institute of Instrumental & Environmental Technology, Otto-von-Guericke-University Magdeburg, Magdeburg, Germany; Natural Environment Research Council, UNITED KINGDOM

## Abstract

There is little information about environmental contamination with antibiotic resistance genes (ARG) in Sub-Saharan Africa, home to about 1 billion people. In this study we measured the abundance of three genes (*sul1*, *sul2*, and *intI1*) used as indicators of environmental contamination with ARGs in the sediments of four urban wetlands in southwestern Nigeria by qPCR. In addition, we characterised the variable regions of class 1 integrons in sulfamethoxazole/trimethoprim (SMX/TRI)-resistant bacteria isolated from the wetlands by PCR and DNA sequencing. The indicator ARGs were present in all wetlands with mean absolute copy numbers/gram of sediment ranging between 4.7x10^6^ and 1.2x10^8^ for *sul1*, 1.1x10^7^ and 1x10^8^ for *sul2*, and 5.3x10^5^ and 1.9x10^7^ for *intI1*. The relative abundances (ARG/16S rRNA copy number) ranged from about 10^−3^ to 10^−1^. These levels of ARG contamination were similar to those previously reported for polluted environments in other parts of the world. The integrase genes *intI1* and *intI*2 were detected in 72% and 11.4% SMX/TRI-resistant isolates, respectively. Five different cassette array types (*dfrA7*; *aadA2*; *aadA1*|*dfrA1*; *acc(6’)lb-cr*|*arr3*|*dfrA27*; *arr3|acc(6’)lb-cr|dfrA27*) were detected among 34 (59.6%) *intI1*-positive isolates. No gene cassettes were found in the nine *intI2*-positive isolates. These results show that African urban ecosystems impacted by anthropogenic activities are reservoirs of bacteria harbouring transferable ARG.

## Introduction

There are increasing concerns about the presence of antibiotic resistant bacteria (ARB) and antibiotic resistance genes (ARG) in the environment [1,2] where they pose special challenges as environmental contaminants. ARB and ARGs can persist and multiply, and ARGs can spread by horizontal gene transfer (HGT) mediated by mobile genetic elements (MGE) within and beyond the original point of occurrence [2], making possible the exchange of resistance traits between environmental bacteria and human pathogens [3]. ARGs and MGE can be enriched in natural ecosystems impacted by human activities such as wastewater discharge [4], solid waste disposal [5,6], metal contamination [7,8], agriculture [9,10,11], aquaculture [12], and drug manufacture [13]. However, very little is known about environmental contamination with ARB and ARGs in developing countries, especially in sub-Saharan Africa. These are critical knowledge gaps since various risk factors favoring the development and spread of antibiotic resistance exist in these environments [14,15].

Aquatic ecosystems are considered important matrices for the release, mixing, persistence and spread of ARB and ARGs associated with horizontally transferable genetic elements [16,17]. In Nigeria, Africa’s most populous nation, wetlands (both coastal and inland) are a key aquatic ecosystem covering about 2.6% of the country’s total land surface [18] with the most extensive being the coastal wetlands found in the southern region including the Lagos and Lekki lagoons and the wetlands of the Niger Delta and Cross Rivers [19]. In addition to the coastal wetlands, several riverine wetlands which are extensively used for livestock grazing, farming and fishing activities are scattered across the country [20]. Presently, existing regulations give little attention to the protection and management of Nigerian wetlands [20], hence, they are constantly exposed to human excreta, raw sewage, untreated wastewater and other pollutants from diverse sources. These forms of anthropogenic impact make the urban wetland ecosystems of Nigeria potential reservoirs of ARB carrying ARGs that might spread to other bacteria through HGT mediated by MGE. However, few studies have investigated pollution of natural wetlands with ARGs and none of those studies emanated from Nigeria.

Integrons are important MGE involved in the capture, mobilization and spread of antibiotic resistance genes in bacterial species [21,22]. The integron platform consists of an integrase gene that can recombine discrete units of circularised DNA known as gene cassettes, primary recombination site *attI*, and a PC promoter that directs transcription of the captured genes [23,24]. Using the sequences of integrase proteins, several classes of integrons have been recognized out of which only a few are important for spreading multidrug resistance in bacteria [25]. Integrons also serve as platform of bacterial evolution [23] and vehicles of gene exchange between the environmental resistome and commensal and pathogenic bacterial species [26] through HGT. Class 1 integrase and sulfonamide resistance genes on has recently been proposed as indicator of pollution by ARB, ARGs and other anthropogenic pollutants [2,27]. In view of this and their association with HGT, an important process for the spread of resistance in environmental reservoirs, it is important to examine genes associated with integrons when investigating antibiotic resistance in the environment.

A few previous studies have reported the detection of clinically relevant ARG in cultivable bacteria isolated from environmental sources in Nigeria [28,29,30,31], but none that we know of used culture-independent quantification to assess the contamination of the Nigerian wetland ecosystems with ARGs. The purpose of this study was to quantify the copy numbers of *sul1*, *sul2*, and class 1 integrase gene (*intI1*) as markers for ARG contamination in the sediments of four polluted wetlands located in Lagos and Ibadan, two of Africa’s most populous cities. Additionally, because integrons are commonly reported in members of the *Enterobacteriaceae* [32] and are found in human impacted ecosystems [25], we investigated the occurrence of class 1 and 2 integrons and their gene cassette contents in 79 sulfamethoxazole/trimethoprim (SMX/TRI)-resistant bacteria of the family *Enterobacteriaceae* isolated from the wetlands.

## Materials and methods

### Sample sites and sample collection

**Fig 1:**
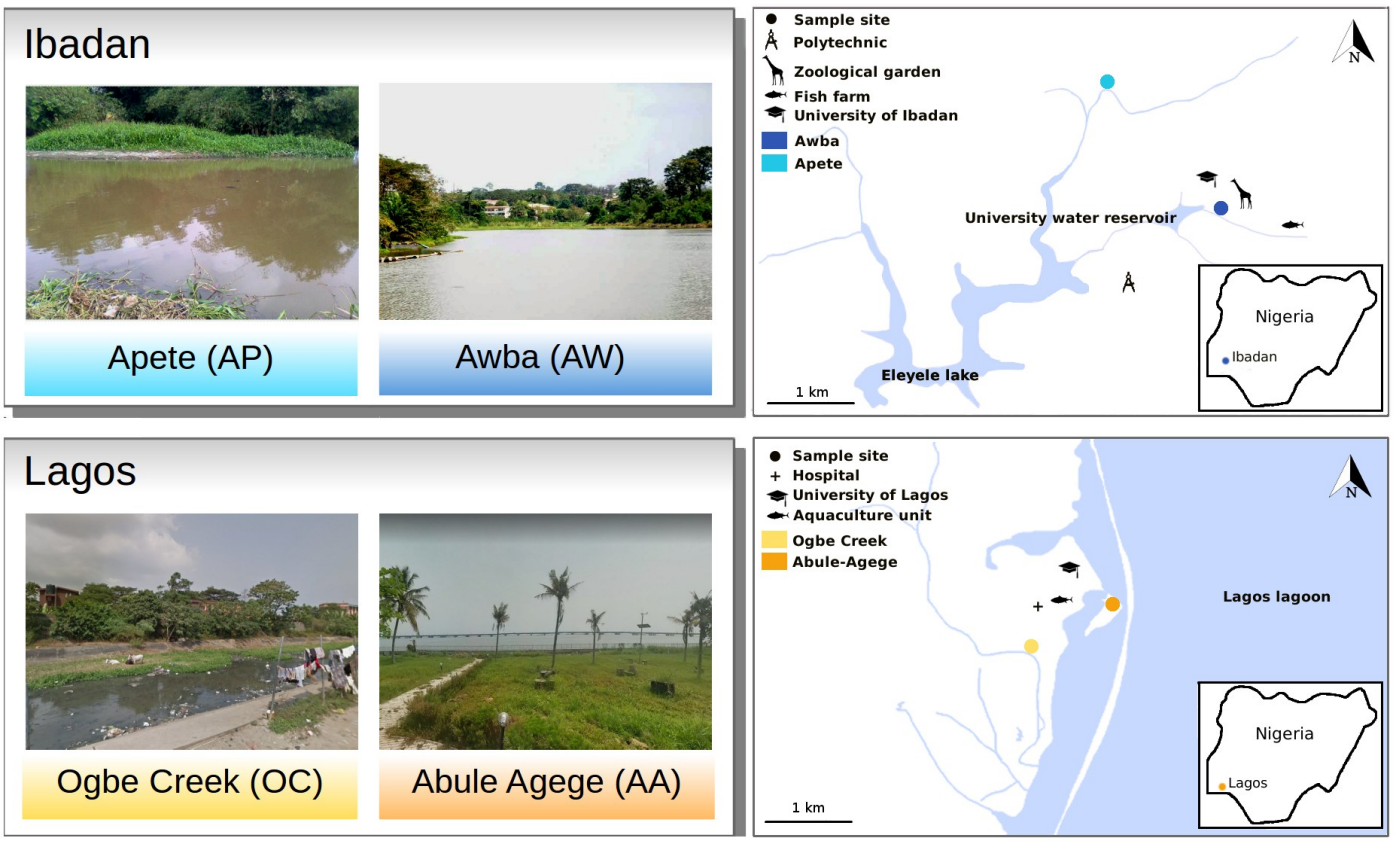
The study sites showing Awba wetland (AW), Apete wetland (AP), Abule-Agege wetland (AA) and Ogbe Creek (OC).

Sediment samples (n = 16) were collected monthly from four wetlands located in Lagos and Ibadan, southwestern Nigeria between October 2014 and January 2015 (Fig 1). The sampling period represented the two major Nigerian seasons “rainy” (sampling in October and November) and “dry” (sampling in December and January). The two wetlands sampled in Ibadan are Awba (AW) (07.4468°N, 03.8763°E) and Apete (AP) wetlands (07.4577°N, 03.8828°E) while in Lagos the two wetlands sampled are Abule-Agege (AA) (06.5145°N, 03.4002°E) and Ogbe Creek (OC) (06.5135°N, 03.3937°E). AW, AP and AA receive untreated wastewater and raw sewage from student hostel facilities of two universities. In addition, the wetlands receive waste streams from fish farms (AW and AA), a Zoological Garden (AW), and seepages from upland solid waste dumpsites (AP and OC). Triplicate sediment samples collected from the upper 1 cm portion of each wetland were pooled to form a composite and stored at -80 °C until processed for analysis.

### DNA extractions

Total sediment community DNA was extracted using FastDNA Spin Kit for soil (MP Biomedicals, Ohio, USA) according to the manufacturer’s instructions. DNA concentrations were estimated with a NanoDrop 1000 (NanoDrop Technologies, Wilmington, DE). Genomic DNA extraction from isolated SMX/TRI-resistant bacteria (see below) was performed by the microwave boiling method [33]. Extracted DNA was stored at –20°C until used.

### Quantitative PCR analysis

The abundances of *sul1*, *sul2*, and *intI1* in the total sediment community DNA of the four wetlands were determined by SYBR-green based real-time PCR with 4 technical replicates per sample. The assay was run on a StepOne Plus Cycler (Applied Biosystems) in a 20 μl reaction mixture. The PCR conditions were 95°C for 2 min, followed by 40 cycles of 95°C for 20 s, 20 s at the respective melting temperatures and 72°C for 20 s. Primers for *sul* genes were those reported by Wang et al. [34] and *intI1* primers those by Mazel et al. [35]. Dilutions of template DNA were used for each sample to compensate for the effect of PCR inhibitors in the samples. The copy number of the 16S rRNA gene in the samples was determined by using Primers 519F and 909R [36] as a measure of total bacterial abundance. Standards were PCR-amplified fragments of the 16S rRNA gene from *Escherichia coli* as well as the *sul* genes and *intI1* from environmental isolates obtained in this study. DNA concentrations of the standards (*c*) were measured by Nanodrop spectrophotometry and the copy number (CN) per gram of wetland sediment was calculated using the relation:

$$\text{CN}=\left(c\times 6.022\times 10^{23}\right)/660\times \text{N}$$

where *c* is the measured DNA concentration (μg/μl) and N is the DNA fragment length in bp.

Efficiency values were 84.5% for *sul1*, 94.1% for *sul2* and 91.3% for *intl1*.

All qPCR data can be found on figshare.com under the title of this report (https://doi.org/10.6084/m9.figshare.7334066.v1).

### Statistical analysis

Measured copy numbers of the genes were log-transformed to normalise the distribution before the statistical significance of the differences in the copy numbers of the genes at each sampling site was determined by analysis of variance (ANOVA) at 5% level of significance (P<0.05). Pearson’s Correlation analysis was used to test the association between measured copy numbers of the 16S rRNA gene, *sul1*, *sul2*, and *intI1*.

### Isolation of SMX/TRI-resistant bacteria

Sediment samples (1 g) were suspended in saline and 100 μl of appropriate dilutions spread on Eosine Methylene blue (EMB) agar plates supplemented with SMX/TRI (56 and 8 μg/ml, respectively). The SMX/TRI combination (co-trimoxazole, Septrin) is commonly used in Nigeria. Bacterial colonies growing on the plates following incubation for 48 h at 30°C were randomly selected, re-streaked on Mueller-Hinton Agar (MHA) plates with SMX/TRI until apparent purity, and the isolates stored in Müller-Hinton broth with 15% (v/v) glycerol at -80°C until further molecular analysis.

The 16S rRNA gene of SMX/TRI-resistant isolates was PCR amplified with universal primers 27F and 1439R [37], amplicons were sequenced (GATC Biotech, Cologne, Germany), and the strains identified via BLASTn queries in the GenBank. The 16S rDNA sequences were deposited in the GenBank database under accession numbers MG859672—MG859730.

### Detection of SMX/TRI resistance genes and integron analysis in SMX/TRI-resistant bacteria

The presence of sulfonamide resistance genes *sul1*, *sul2* and *sul3* and the most commonly found trimethoprim resistance genes *dfrA1*, *dfrA5*, *dfrA7*, *dfrA12* and *dfrA17* was investigated in the SMX/TRI-resistant bacteria by PCR as described [34,38] using either QIAGEN *HotStarTaq* Mastermix (QIAGEN GmbH, Hilden, Germany) (*sul1* and *sul2*), KAPA HiFi *HotStart* PCR Kit (KAPA Biosystems, Boston, USA) (*sul3*), *Dream Taq Green* PCR Mastermix (2x) (ThermoFisher Scientific, Waltham, USA) (*dfrA1*), or Kappa 2G Robust PCR kit (KAPA Biosystems, Boston Massachusetts, USA) (*dfrA5*, *dfrA7*, *dfrA12* and *dfrA17*). The presence of integrase genes *intI1*, *intI2* and *intI3* was queried for by PCR and the variable region of class 1 integron characterised by PCR and Sanger sequencing as described elsewhere [39]. The diversity of promoters associated with detected class 1 integrons were characterised by Sanger sequencing as already described [40,41]. The sequenced sections of the class I integrons were deposited at GenBank under accession numbers MK093863 –MK093896.

### Literature survey for qPCR data on *sul* and *intI1* genes in aquatic sediments

In order to compare abundances of *sul* and *intI1* genes in the Nigerian wetlands with values reported in the literature we searched the ScienceDirect and Scopus databases using the keywords “antibiotic resistance genes” and “wetlands”. We chose studies that reported the genes’ relative abundances for sediments in natural aquatic environments. Eleven studies from China, Pakistan, Poland, USA, Sweden and Switzerland but none from Africa met our criteria [42–53]. In cases where more than one season was examined in a study, we extracted the values for summer in order to be closer to the climatic conditions in Nigeria. When data from different sample sites was provided, we took the highest values.

## Ethical statement

There are no specific permits required for sample collection in the field studies. The wetlands not protected and not privately owned, hence there are no regulations that restrict collection of sediment samples for research purposes from the four wetlands. The field study does not involve any endangered or protected species, only sediment samples were taken from the sites.

## Results and discussion

### Abundances of *intI1* and *sul* genes show medium to heavy pollution with antibiotic resistance genes in four urban Nigerian wetlands

**Fig 2:**
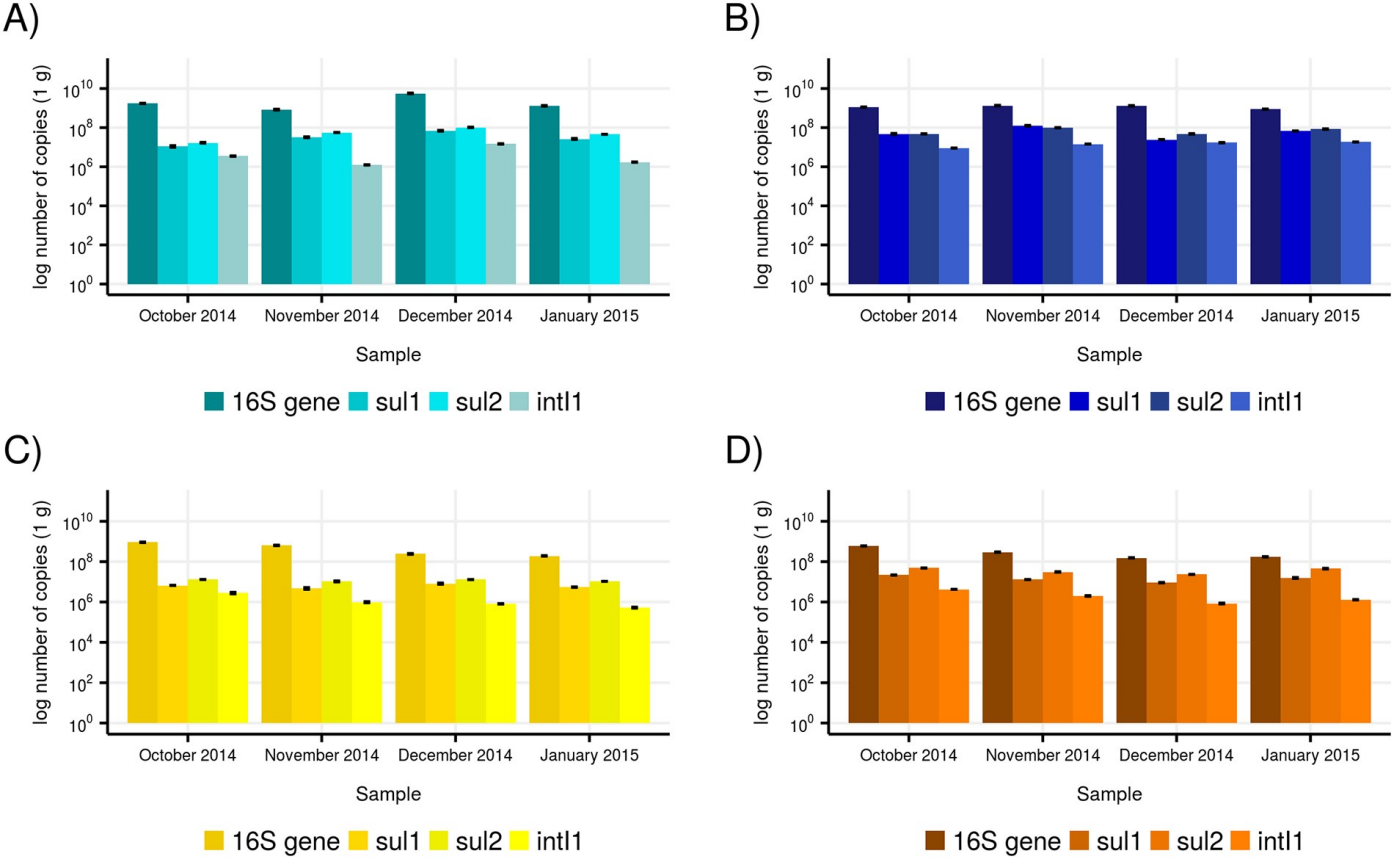
Mean absolute abundances of 16S rRNA, class 1 integrase (*intI1*) and sulfonamide resistance (*sul1* and *sul2*) genes in Awba (A), Apete (B), Abule-Agege (C) and Ogbe Creek (D) for October 2014, November 2014, December 2014 and January 2015.

The *intI1*, *sul1*, and *sul2* genes were detected in all the samples analysed. The mean absolute copy numbers/gram of wetland sediment of the genes and the 16S rRNA gene are shown in Fig 2. The mean copy numbers of 16S rRNA gene ranged from 1.6x10^8^ (December 2014, AA) to 5.7x10^9^ (December 2014, AP) copies per g of sediment. The *intI1* gene was detected in the wetlands at values ranging from 5.3x10^5^ (January 2015, OC) to 1.9x10^7^ (January 2015, AW) copies/gram sediment. These abundances were in the range reported for aquatic sediments in other regions of the world [25]. It should be mentioned that the respective studies cited in [25] used various primers for the quantification of the *intI1* gene, which hampers the comparative assessment of the levels of *intI1* contamination in the respective contaminated sites.

The copy number/gram sediment of *sul1* in the four wetlands ranged from 4.7x10^6^ (November 2014, OC) to 1.2x10^8^ (November 2014, AW). The absolute abundances of *sul2* was higher than that of *sul1* at 1.1x10^7^ (November 2014, OC) to 1x10^8^ (December 2014, AP) copy number/gram sediment. There was no universal trend regarding the ratio of *sul1* to *sul2* in sediments. Greater abundance of *sul2* over *sul1* was found by Luo et al. [43] similar to what we found in our study sites, similar abundance of both genes were measured by Jiang et al. [54] and Lu et al. [55], while a higher abundance of *sul1* was reported by Gao et al. [56] and Koczura et al. [48].

To the best of our knowledge, there are no similar studies of ARG contamination of the Nigerian environment available for the comparative analysis of our results. Overall, the absolute levels of *sul* contamination in the wetlands of this study are in the upper range of values reported for wetlands and river sediments in other parts of the world [42,46,48,51,55–59]. The abundance of the *sul* genes as well as those of *intI1* were significantly different (p<0.01) across the four wetlands. In particular the AW and AA sites had *sul* abundances mirroring those of heavily contaminated sites elsewhere [42,60]. These differences in ARG abundance in the four wetlands correlates with the different anthropogenic activities associated with the wetlands. AW receives direct input of untreated domestic wastewater, discharges from a fish farm and a Zoological Garden. The only known point source of anthropogenic input identified for OC, the wetland with the lowest ARG abundance, is an upland solid waste dumpsite which appears to be relatively new at the time of sample collection. There was no significant difference in the abundance of the ARGs measured for the samples collected in the rainy season (October and November) and those collected in the dry season (December and January).

The absolute abundance of the *sul* genes showed positive correlation with the copy number of *intI1* (*P<*0.01 or *P<*0.05). The correlation coefficient of *sul1* (0.794) is slightly higher than that of *sul2* (0.729). This is expected as *sul1* is typically located in the 3’ conserved segments of class 1 integrons [32]. However, the mean absolute copy number of *intI1* in all the samples was generally lower than the mean absolute copy number of *sul1*, which is similar to what has been reported in previous studies [47,48].

It should be mentioned that the *intI1*–directed primers [35] used in our study did not exclusively target clinical *intI1* and thus may have resulted in an overestimation of anthropogenic pollution with the clinical class I integron. Yet that overestimation might not be substantial. Antelo et al. [61] used the same primers as in our study in a PCR-based survey on integron diversity with samples from King George Island, Antarctica, and found a prevalence rate of 42% of clinical class I integrons. On the other hand, the *sul–*targeting primers used here were designed based on the sequence of a single allele of the respective *sul* gene. This could have resulted in an underestimation of the *sul* gene abundances in our study sites as a recent study has shown that a wide variety of both clinical and non-clinical *sul* alleles are present in polluted natural ecosystems [62].

**Fig 3:**
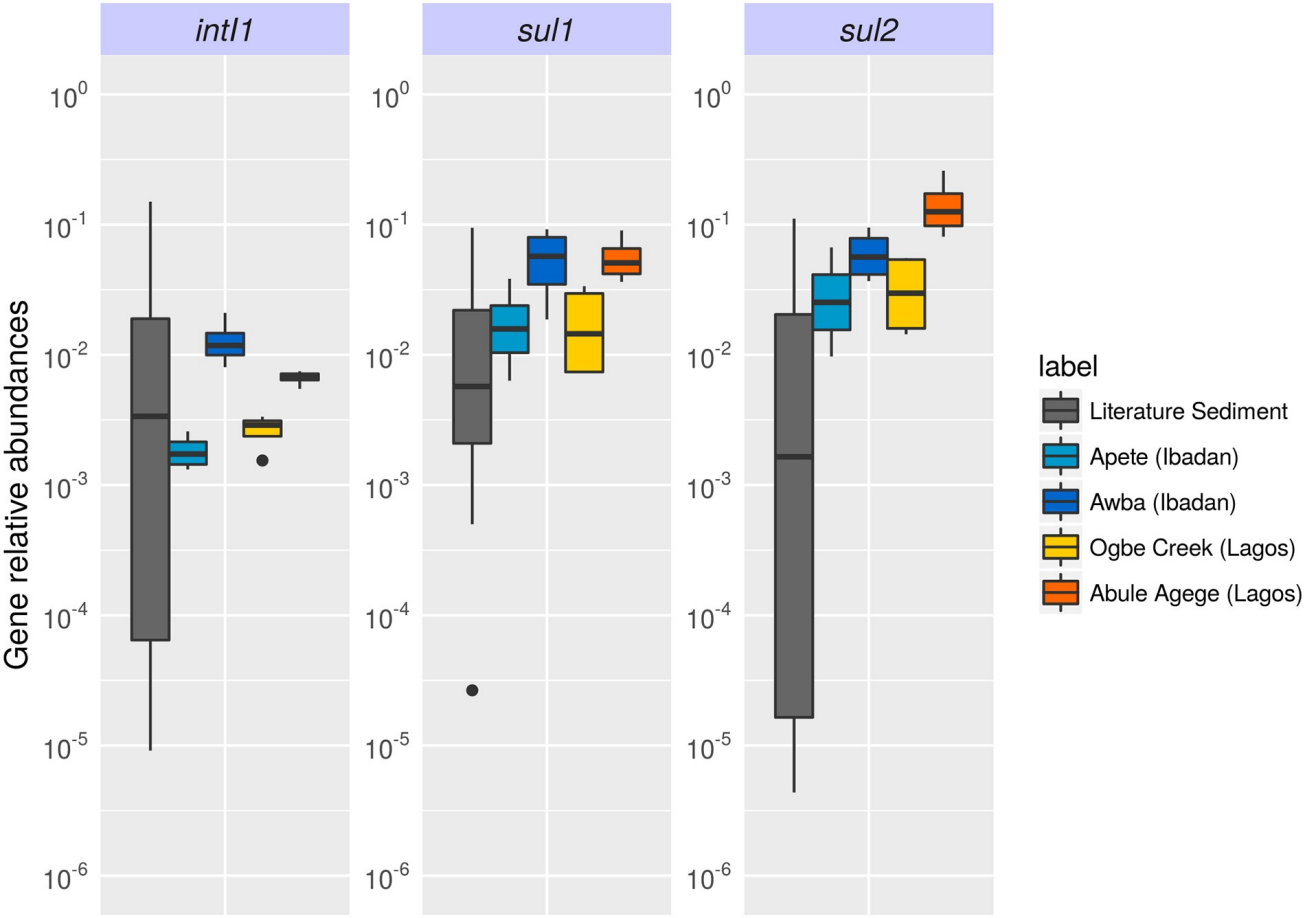
Relative abundance of indicator ARGs from our study sites (coloured bars) compared to similar sites in China [43,46,47,49–51], Pakistan [52], Poland [48], Sweden [45], Switzerland [44] and USA [42] (grey bars).

Essentially the same picture of the level of contamination of the wetlands investigated here is apparent from the relative abundance of *intI1*, *sul1*, and *sul2* over 16S rRNA gene copy numbers (Fig 3). The relative abundance of *intI1* was similar to the values reported for aquatic sediments elsewhere [25], while the relative abundance of *sul* genes indicate heavy contamination, especially of the AW and AA sites. These relative abundances are similar to values found at a pig farm where SMX was used [63], mariculture sites in China [64], and water bodies around a drug formulation facility in Pakistan [52].

### SMX/TRI genes and integrons are prevalent in bacterial isolates from the wetlands

To understand the genetic context of the SMX/TRI resistance genes and identify other transferable genes associated with detected ARG markers, SMX/TRI-resistant *Enterobacteriaceae* were isolated from the four wetlands and screened by PCR for sulfonamide resistance genes *sul1*, *sul2*, *sul3*, the trimethoprim resistance genes *dfrA1*, *dfrA5*, *dfrA7*, *dfrA12*, *dfrA17*, and integrons class 1, 2 and 3. Despite its obvious limitations, data from cultivable bacteria are still important in the study of antibiotic resistance. They provide insight into the phenotypic and genotypic characteristics of bacteria isolates and are therefore integral to national and international antibiotic resistance surveillance and tracking efforts. Further, they also provide full insight into the resistome of natural ecosystems and the link between antibiotic resistances detected in the environment and clinical settings when combined with data generated from high throughput culture-independent methods [65].

Out of a total of 79 strains (AW = 23, AP = 19, AA = 17, OC = 20), 50 strains (63%) carried at least one of the SMX/TRI resistance genes tested for. 16S rRNA gene sequencing identified these 50 isolates as *Citrobacter* sp. (27), *Enterobacter* sp. (16), *Escherichia* sp. (3), *Pseudomonas* sp. (3), and *Achromobacter* sp. (1). Some of the isolates shared identical 16S rRNA sequences, however, the pattern of genes detected in the isolates were either different or where the isolates shared identical pattern of genes, they were isolated at different time periods. s*ul1* was the most frequently detected gene, found in 40/79 (51%) strains (AW = 18, AP = 16 and AA = 6), followed by *sul2* found in 32/79 (41%) strains (AW = 10, AP = 10, AA = 10, OC = 2), and *sul3* found in only 1 strain from AW also carrying *sul1* and *sul2*. *dfrA1*, *dfrA12* and *dfrA7* were detected in 1, 2 and 10 isolates, respectively, *dfrA5* and *dfrA17* were not detected in any of the isolates. The gene *dfrA7* occurred with *sul1* in 4 isolates and with *sul1* and *sul2* in five isolates.

*intI1* and *intI2* were detected in 57 (72%) and 9 (11.4%) SMX/TRI-resistant isolates respectively, including eight bacteria (*intI1 =* 7, *intI2 =* 1) where none of the SMX/TRI genes were found. *intI*3 was not detected in any of the bacterial strains investigated. This is consistent with previous reports that class 1 and 2 integrons are the most frequently detected integrons in Gram negative bacteria [66] with class 2 integrons detected less frequently than class 1 integrons.

### Characterisation of integrons gene cassettes

Because of the important role of integrons in the spread of resistance in natural ecosystems, we carried out PCR analysis together with DNA sequencing of the variable regions of detected *intI1* and *intI2* among isolated SMX/TRI-resistant bacteria to identify additional ARGs that may potentially spread by HGT. In all *intl*2-positive isolates, the primer pair attl2 and orfX failed to yield any useful amplification product in all cases indicating that they are likely to be novel integrons or integrons without gene cassettes. In contrast, variable regions containing gene cassettes were amplified in 34 of 57 (59.6%) *intI1*-positive isolates including three isolates where none of the tested SMX/TRI genes was detected. The remaining *intI1*-positive isolates either lack gene cassettes or might be derivatives of *Tn*5090-like class 1 integrons that lack the 3’ conserved end [67,68]. Seven different types of genes arranged in five different arrays were found within the variable regions of the class 1 integrons in the 34 isolates (Table 1). The genes encode resistance to trimethoprim (*dfrA1*, *dfrA7*, *dfrA27*), aminoglycosides (*aadA1*, *aadA2*), rifampicin (*arr-3*) and fluoroquinolones (*acc(6’)-lb-cr*). The *dfrA* genes were the most frequently detected gene cassettes occurring in (82%) of the gene cassette-bearing isolates. This is similar to previous reports by Lin et al. [69] and corroborated previous reports that class 1 integrons are important for the dissemination of SMX/TRI resistance genes [70].

**Table 1:** Resistance genes, class 1 integron gene cassettes and promoter types in SMX/TRI-resistant bacteria from the four wetlands in Ibadan and Lagos, Nigeria.

Isolates	Phylogenetic placement	Date of isolation	Resistance genes/Integrons	Integron gene cassettes	Promoter Types
**Awba Wetland**					
PAW1-6	*Citrobacter* sp.	October 2014	*sul1*, *sul2*, *sul3*, *intI1*, *intI2*	*aadA1|dfrA1*	PcH1-P2
PAW1-4	*Citrobacter* sp.	October 2014	*sul1*, *intI1*, *intI2*	*acc(6’)lb-cr|arr3| dfrA27*	PcW-P2
PAW2-4	*Enterobacter* sp.	November 2014	*sul1*, *intI1*	*aadA2*	PcW-P2
EAW2-1	*Citrobacter* sp.	November 2014	*sul1*, *sul2*, *intI1*	*aadA2*	PcW-P2
EAW2-3	*Enterobacter* sp.	November 2014	*sul1*, *intI1*	*aadA2*	PcW-P2
PAW2-6	*Enterobacter* sp.	November 2014	*sul1*, *sul2*, *intI1*	*aadA2*	PcW-P2
PAW3-1	*Enterobacter* sp.	December 2014	*sul1*, *sul2*, *intI1*	*acc(6’)lb-cr|arr3| dfrA27*	PcW-P2
PAW3-3	*Citrobacter* sp.	December 2014	*sul1*, *sul2*, *intI1*, *intI2*	*acc(6’)lb-cr|arr3| dfrA27*	PcW-P2
PAW4-5	*Enterobacter* sp.	January 2015	*sul1*, *dfrA7*, *intI1*	*dfrA7*	PcW-P2
EAW4-6	*Citrobacter* sp.	January 2015	*sul1*, *dfrA1*, *intI1*	*aadA1|dfrA1*	PcH1-P2
PAW4-4	*Citrobacter sp*	January 2015	*sul1*, *intI1*, *intI2*	*acc(6’)lb-cr|arr3| dfrA27*	PcW-P2
**Apete Wetland**					
EAP1-2	*Enterobacter* sp.	October 2014	*sul1*, *sul2*, *intI1*	*acc(6’)lb-cr|arr3| dfrA27*	PcW-P2
EAP1-3	*Citrobacter* sp.	October 2014	*intI1*	*acc(6’)lb-cr|arr3| dfrA27*	PcW-P2
EAP1-5	*Pseudomonas* sp.	October2014	*sul1*, *sul2*, *intI1*	*acc(6’)lb-cr|arr3| dfrA27*	PcW-P2
PAP2-5	*Citrobacter* sp	November 2014	*intI1*	*acc(6’)lb-cr|arr3| dfrA27*	PcW-P2
PAP2-6	*Citrobacter* sp.	November 2014	*sul1*, *sul2*, *dfrA7*, *intI1*	*dfrA7*	PcW-P2
EAP2-1	*Citrobacter* sp.	November 2014	*sul1*, *dfrA7*, *intI1*	*dfrA7*	PcH1-P2
EAP2-3	*Citrobacter* sp.	November 2014	*sul1*, *sul2*, *dfrA7*, *intI1*	*dfrA7*	PcW-P2
PAP2-2	*Citrobacter* sp.	November 2014	*sul1*, *sul2*, *dfrA7*, *intI1*	*dfrA7*	PcW-P2
PAP2-3	*Citrobacter* sp.	November 2014	*sul1*, *sul2*, *dfrA7*, *intI1*	*dfrA7*	PcW-P2
PAP4-16	*Citrobacter* sp.	January 2015	*sul1*, *dfrA7*, *intI1*	*dfrA7*	PcW-P2
PAP4-3	*Citrobacter* sp.	January 2015	*sul1*, *sul2*, *dfrA7*, *intI1*	*dfrA7*	PcW-P2
EAP4-6	*Achromobacter* sp.	January 2015	*dfrA7*, *intI1*	*dfrA7*	PcH1-P2
EAP1-1	*Citrobacter* sp.	January 2015	*sul1*, *sul2*, *intI1*	*acc(6’)lb-cr|arr3| dfrA27*	PcW-P2
PAP4-5	*Citrobacter* sp.	January 2015	*sul1*, *intI1*	*acc(6’)lb-cr|arr3| dfrA27*	PcW-P2
EAP4-1	*Citrobacter* sp.	January 2015	*sul1*, *intI1*	*acc(6’)lb-cr|arr3| dfrA27*	PcH1-P2
PAP4-1	*Citrobacter* sp.	January 2015	*sul1*, *sul2*, *intI1*	*acc(6’)lb-cr|arr3| dfrA27*	PcW-P2
EAP4-5	*Pseudomonas* sp.	January 2015	*sul1*, *sul2*, *intI1*	*acc(6’)lb-cr|arr3| dfrA27*	PcH1-P2
**Abule-Agege**					
PAA1-2	*Escherichia* sp.	October 2014	*sul1*, *sul2*, *intI1*	*arr3| acc(6’)lb-cr|dfrA27*	PcW-P2
PAA2-7	*Citrobacter* sp.	November 2014	*sul1*, *intI1*	*aadA2*	PcW-P2
PAA2-8	*Citrobacter* sp.	November 2014	*sul1*, *sul2*, *intI1*	*aadA2*	PcW-P2
EAA3-5	*Citrobacter* sp.	December 2014	*drfA12*, *intI1*	*arr3| acc(6’)lb-cr|dfrA27*	PcH1-P2
PAA4-1	*Citrobacter* sp.	January 2015	*sul1*, *sul2*, *intI1*	*arr3| acc(6’)lb-cr|dfrA27*	PcW-P2
EAA4-2	*Enterobacter* sp.	January 2015	*intI1*	*dfrA7*	PcH1-P2

ND: Not Detected, PcW: Weak Promoter, PcH1: Hybrid promoter 1, P2: Inactive promoter 2

Type 1 (769 bp) gene cassettes shared 99% nucleotide sequence identity with class 1 integron from *Acinetobacter baumannii* 607460 (EU340417.1) containing *dfrA7* and was found in 10 isolates (AW = 1, AP = 8 and AA = 1). Type 2 (2.2 kb) cassette array shared 99% sequence identity with class 1 integron of *Citrobacter freundii* strain S12 (KR259319.1) containing the plasmid mediated quinolone resistance gene *acc(6’)lb-cr*, the rifampicin resistance gene *arr3* and the trimethoprim resistance gene *dfrA27* and was found in 16 isolates (AW = 4, AP = 9 and AA = 3). The order of arrangement of the Type 2 gene cassettes varied. In the isolates from Ibadan (AW and AP) it was *acc(6’)lb-cr|arr3|dfrA27* (Type 2a) while in the isolates from Lagos (AA) it was *arr3|acc(6’)lb-cr|dfrA27* (Type 2b). Type 3 (1.5kb) array, found in two isolates from AW, shared 95% identity with class 1 integron of *E*. *coli* strain Ec171 (GU590934.1) containing the streptomycin/streptogamin resistance gene *aadA1* and trimethoprim resistance gene *drfA1*, while Type 4 (730 bp) cassette array shared 99% sequence identity with the streptomycin/streptogamin resistance gene *aadA2* on the class 1 integron of *Proteus mirabilis* strain NF991579 (HQ880254.1). Type 4 was found in six isolates from AW (n = 4) and AA (n = 2). Four cassette array types (1, 2a, 3 and 4) were represented in bacteria from AW, while isolates from AP carried Type 1 and 2a, and those from AA harbored Types 1, 2b and 4 arrays respectively, supporting previous reports that gene cassette populations can vary markedly even within small physical distances [71] with type of selection pressure playing important roles in shaping cassette diversity [68].

All the array types reported in this study have been previously reported in the literature. Array types 1 and 3 in particular have been reported widely in bacteria from human clinical sources, aquaculture, farm animals, rivers and hospital wastewater in Nigeria and Ghana [29,72,73], Central African Republic [74], Egypt [75], Europe [76–83], China [84–86], Brazil [87], Canada [54] and US [88]. The Type 1 cassette (*dfrA7*) was found associated with a widely disseminated Tn*21*-type transposon in Nigeria and Ghana [72]. Cassette array Type 4 has also been reported in bacteria from wastewater in Mozambique [89], rivers in Nigeria [90] and China [86], *E*. *coli* isolates from Nigeria [72] and an on-farm bio-purification plant [9]. Only recently, type 2a cassettes was reported in six bacteria species (*Klebsiella pneumoniae*, *Acinetobacter nosocomialis*, *C*. *freundii*, *Pantoea agglomerans*, *Stenotrophomonas maltophilia* and *Staphylococcus xylosus*) isolated from American eel (*Anguilla rostrata*) and pond water in Fujian Province, China [69]. We additionally detected this array type in *Pseudomonas* and *Enterobacter* spp. confirming its potential for wide dissemination among bacterial species.

### Diversity of promoters of class 1 integrons

We analysed the promoter types in the *intI1*-positive isolates. Two different Pc promoter types, the weak promoter PcW and the Hybrid Type 1 promoter PcH1 were detected in 26 and 8 isolates, respectively, of 34 *intI1*-positive isolates. In all cases, the detected promoters were associated with an inactive second promoter type P2 where the -35 and -10 hexamers of the respective promoters were separated by 14 bp instead of the 17 bp expected with an active P2 [91]. Jové et al. [40], Vinue et al. [41] and Moura et al. [92] similarly reported PcW as the most frequently detected promoter type in bacteria from clinical and environmental sources. Since promoter strength and integrase excision activity are inversely correlated, thus affecting the propensity for the dissemination of resistance genes [40]; the prevalence of weak PC variants in this study suggests that there is a high propensity for further dissemination of the antibiotic resistance genes carried on the class 1 integrons among bacteria in the wetlands.

In summary, indicator ARGs *sul1*, *sul2*, and *intI1* were detected in all four Nigerian wetlands at high absolute and relative (vs. the 16S rRNA gene) gene frequencies. Class 1 and 2 integrons were found in 72% and 11.4% of 79 culturable bacteria from the wetlands with five gene cassette arrays containing 7 different resistance genes found in 59.6% of detected class 1 integrons. No gene cassette was detected in the class 2 integrons. Our results demonstrate that the Nigerian wetlands are contaminated with ARGs at levels similar to those usually reported for highly contaminated sites in other parts of the world. Although only a limited number of ARGs and wetlands were selected, this study provides the first data on the status of ARG contamination in any Nigeria’s aquatic ecosystem and suggested a role for anthropogenic activities in environmental contamination with ARGs. Larger studies are needed for an overall evaluation of the magnitude of ARG contamination in the Nigerian aquatic ecosystem and its associated risks.
